# Microbial reduction of prebagged human plasma using 405 nm light and its effects on coagulation factors

**DOI:** 10.1186/s13568-024-01725-0

**Published:** 2024-06-06

**Authors:** Caitlin F. Stewart, Preston McGoldrick, John G. Anderson, Scott J. MacGregor, Chintamani D. Atreya, Michelle Maclean

**Affiliations:** 1 The Robertson Trust Laboratory for Electronic Sterilisation Technologies (ROLEST), Department of Electronic & Electrical Engineering, University of Strathclyde, Royal College Building, 204 George Street, Glasgow, UK; 2 Department of Biomedical Engineering, University of Strathclyde, Glasgow, UK; 3 Office of Blood Research and Review, Center for Biologics Evaluation and Research (CBER), Food and Drug Administration, Silver Spring, MD USA

**Keywords:** Human plasma, Blood transfusion, Pathogen reduction, Violet-blue light, 405-nm

## Abstract

Bacterial contamination is the most prevalent infectious complication of blood transfusion in the developed world. To mitigate this, several ultraviolet light-based pathogen reduction technologies (PRTs), some of which require photo-chemicals, have been developed to minimize infection transmission. Relative to UV light, visible 405-nm light is safer and has shown potential to be developed as a PRT for the *in situ* treatment of *ex vivo* human plasma and platelet concentrates, without the need for photo-chemicals. This study investigates the effect of 405-nm light on human plasma, with focus on the compatibility of antimicrobial light doses with essential plasma clotting factors. To determine an effective antimicrobial dose that is compatible with plasma, prebagged human plasma (up to 300 mL) was seeded with common microbial contaminants and treated with increasing doses of 405-nm light (16 mW cm^−2^; ≤ 403 J cm^−2^). Post-exposure plasma protein integrity was investigated using an AOPP assay, *in vitro* coagulation tests, and ELISA-based measurement of fibrinogen and Protein S. Microbial contamination in 300 mL prebagged human plasma was significantly reduced (P ≤ 0.05) after exposure to ≤ 288 J cm^−2^, with microbial loads reduced by > 96.2%. This dose did not significantly affect the plasma protein quality parameters tested (P > 0.05). Increased doses (≥ 345 J cm^−2^) resulted in a 4.3% increase in clot times with no statistically significant change in protein activity or levels. Overall, this study has demonstrated that the effective microbicidal 405 light dose shows little to no negative effect on plasma quality.

## Introduction

Donor screening and advancements in infectious marker testing have significantly improved blood safety, however the risk of bacterial contamination remains, and a variety of other infectious agents including newly emerging viruses and parasites continue to threaten blood transfusion safety (Brecher et al. 2003; Gehrie et al. 2019). Recent advancements have led to the development of a selection of chemical- and/or optical-based pathogen reduction technologies (PRTs) which proactively prevent and control infection transmission by inactivating blood-borne pathogens before transfusion (Gehrie et al 2019; Marschner and Dimberg 2019). These PRTs provide high antimicrobial efficacy and have shown to be clinically safe, however several studies have reported reductions in post-treatment blood components’ quality and functionality, with emphasis on the functional impediment of coagulation factors in human plasma, and the requirement for more frequent PC transfusions in patients receiving PRT-treated products (Escolar et al. 2022; Gehrie et al. 2019; Hamzah et al. 2018; Klein 2005; Magron et al. 2018; Schlenke et al. 2014; Seltsam 2017). Further, use of ultraviolet (UV) light wavelengths and chemical photosensitizers have operational disadvantages as treatments require bag transfers and/or additive removal stages which increases processing times.

The ideal PRT would provide an optimal balance of antimicrobial activity and blood product compatibility, whilst being relatively inexpensive and simple to implement. Our research focuses on the development of a novel alternative PRT for the treatment of blood transfusion products using visible, violet-blue 405-nm light. The inactivation mechanism of 405-nm light is driven by the photo-excitation process of intracellular porphyrin molecules within exposed microbial cells, which in turn generates reactive oxygen species (ROS) capable of widespread oxidative damage and cell death (Godley et al. 2005; Guffey et al. 2006; Hamblin et al. 2005; McKenzie et al. 2016; Ramakrishnan et al. 2016). The widespread inactivation capabilities of 405 nm light, reviewed by Tomb et al. (2018) are well established, with studies demonstrating efficacy against a range of vegetative bacteria, bacterial endospores, fungi, yeast, and under certain conditions, viruses (Enwemeka et al. 2008; Maclean et al. 2013, 2009; Murdoch et al. 2013, 2012; Tomb et al. 2017).

To date, 405-nm light has successfully demonstrated its potential as a PRT for human plasma and platelet concentrates suspended in plasma (PCs) using a range of bacteria, a yeast and more recently, the blood-borne parasites *Trypanosoma cruzi* and *Leishmania donovani*, and an enveloped virus, human immunodeficiency virus-1 (HIV-1) (Jankowska et al. 2020; Kaldhone et al. 2024; Lu et al. 2020; Maclean et al. 2020, 2016; Ragupathy et al. 2022; Stewart et al. 2020). Proof-of-concept studies also showed the capacity for 405-nm light to reduce pathogens in human plasma *in situ* within the transfusion bag. This procedure also eliminates the need for additional photosensitizers (Lu et al. 2020; Maclean et al. 2020, 2016).

A recent report utilising low volume (250 µL) plasma samples demonstrated broad-spectrum microbial inactivation, while preserving plasma protein integrity (Stewart et al. 2020), however, demonstrable scale-up of the treatment is required to ensure there is potential for this technology to be utilised for pathogen reduction of prebagged volumes. Additionally, to date, the effect of 405 light on coagulation factors present in plasma have not been evaluated for *in vitro* functional parameters, which is a critical step for any PRT to be successful (for both plasma, and PCs suspended in plasma). Accordingly, the primary focus of this study is on assessing the light effect on microbial reduction of prebagged plasma (up to 300 mL volumes), and assessment of the *in vitro* functional parameters of plasma coagulation factors following treatment with antimicrobial doses.

## Materials and methods

### Human plasma

**Fig. 1:**
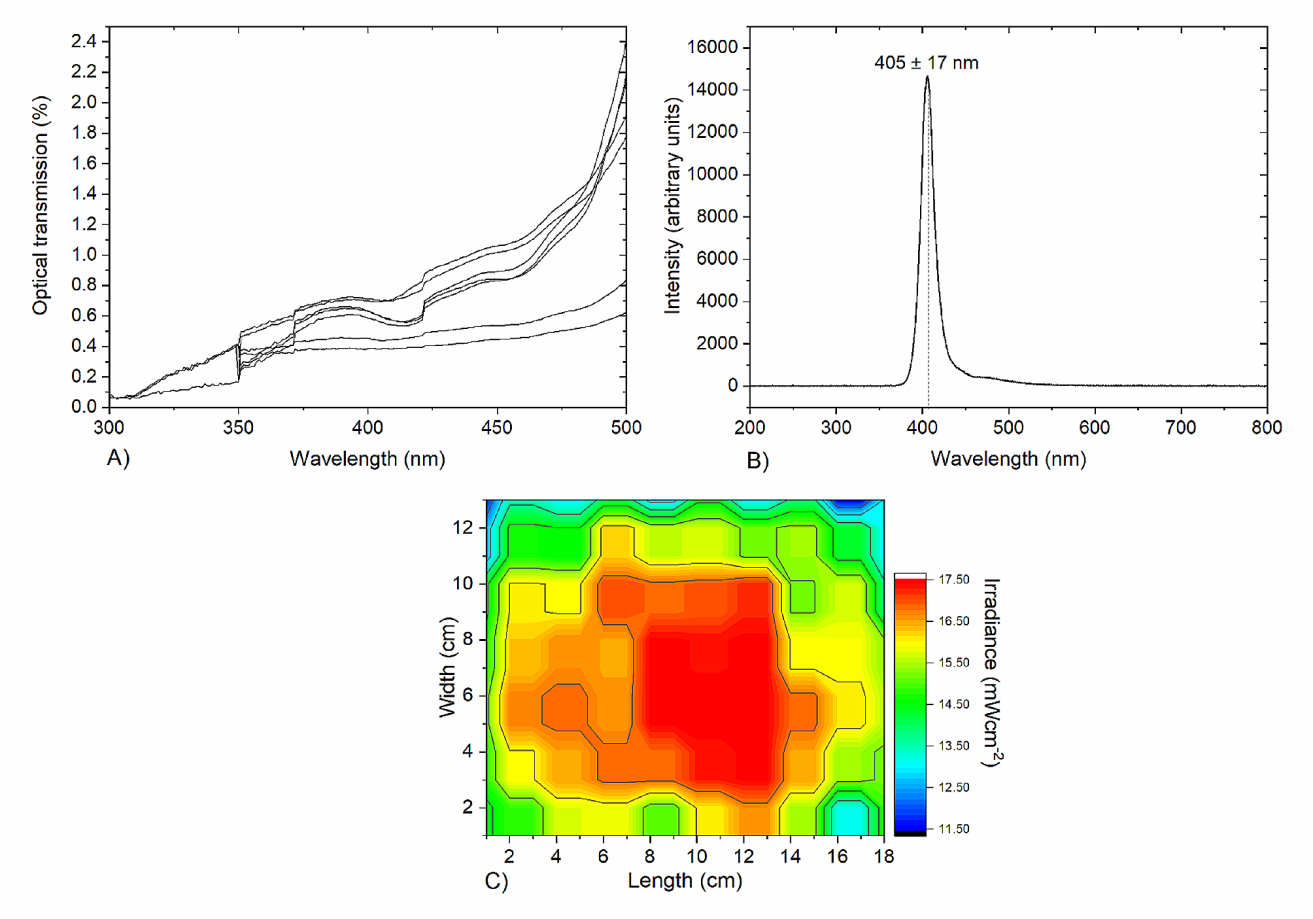
Optical characterization of human plasma, and the 405-nm light exposure system for the treatment of prebagged human plasma. **a** Optical transmission of human plasma measured between 300 and 500 nm using a UV–Vis spectrophotometer. Analysis shows the slight variation in transmissibility of samples from different batches (n = 8). **b** Optical emission spectrum of the 405-nm LED arrays used in the light unit, captured using a high-resolution spectrometer (HR4000, Ocean Optics Inc, Germany) and SpectraSuite software (Version 2.0.151). **c** Irradiance profile across the blood bag surface with an average irradiance of ~ 16 mW cm^−2^ measured, taking account of the loss of light transmission through the blood bag material (plotted using OriginPro 2018 software)

Human plasma (TCS Biosciences Ltd (UK)) was stored at −18 °C and thawed at ambient room temperature for ∼ 2 h prior to experimentation. The transmissibility of human plasma was found to be in the region of 0.38–0.7% at 405 nm (Fig. 1A) using UV–Vis spectrophotometry (Biomate-5, Thermo Spectronic). For light exposures, 100 and 300 mL human plasma was suspended in 150 and 450 mL blood bags (Grifols, UK).

### Microbial cultures and artificial contamination of human plasma

The organisms used in this study were: *Staphylococcus aureus* NCTC 4135, *Staphylococcus epidermidis* LMG 10273 (Gram-positive bacteria); *Escherichia coli* NCTC 9001, *Pseudomonas aeruginosa* NCTC 9009, and *Acinetobacter baumannii* LMG 1041 (Gram-negative bacteria); and *Candida albicans* DSM 1386 (a yeast). Cultures were obtained from the National Collection of Type Cultures (NCTC, Colindale, UK), Belgian Coordinated Collections of Microorganisms (LMG, Bruxelles, Belgium) and the Leibniz Institute DSMZ-German Collection of Microorganisms and Cell Cultures (DSM, Braunschweig, Germany). Organisms were cultured at 37 °C for 24 h under rotary conditions (120 rpm), in nutrient broth (Oxoid Ltd., UK), except for *S. epidermidis* and *C. albicans* which were cultured in tryptone soya broth, and malt extract broth with 0.1% yeast extract (Oxoid Ltd., UK), respectively. Broths were centrifuged at 3939×*g* for 10 min and the cell pellets re-suspended and serially-diluted in phosphate buffered saline (PBS; Oxoid Ltd, UK) to 10^5^ colony-forming units per millilitre (CFU mL^−1^). This microbial suspension was then used to spike the prebagged human plasma to achieve a contamination level of ∼ 10^3^ colony-forming units per millilitre (CFU mL^−1^) in 100 mL volumes of prebagged plasma. Scaled up testing using 300 mL prebagged plasma was then conducted using *S. aureus*, *E. coli* and *C. albicans* (representative of a Gram positive, Gram negative, and yeast species, respectively), and were prepared using the same procedure.

### Violet-blue 405-nm light system

Exposure of prebagged human plasma was conducted using a prototype light treatment system (US Patent Application No. 62/236, 706, 2015), composed of an incubator shaker with a top-mounted light source. The light source contained light emitting diode (LED) arrays (LZ4-00UB00-U7, Osram LED Engin, USA), with a centre wavelength of 405 nm (∼ 17 nm FWHM), shown in Fig. 1B, powered in parallel by a 15 V LED driver (Model HLG-80H-15, Mean Well, Netherlands). The 405-nm light source was held in a fixed position, 12 cm above the base of the shaker plate, where the plasma bags were horizontally positioned. During exposures, plasma bags were held at 22 °C under continuous agitation, 84 rpm). The optical profile of the light distribution across the blood bag, measured using a radiant power meter and photodiode detector (LOT-Oriel Ltd, USA), is shown in Fig. 1C. An average irradiance of ~ 16 mW cm^−2^ was measured across the bag surfaces, taking into account a 26% reduction in irradiance due to transmission through the blood bag material. Prebagged human plasma was treated with this fixed irradiance for treatment times up to 7 h, with the applied dose calculated using the equation:

1 $$ \begin{aligned} Dose\left( {{\text{J}}{\mkern 1mu} {\text{cm}}^{{ - 2}} } \right) = & Average\,irradiance\left( {{\text{W}}{\mkern 1mu} {\text{cm}}^{{ - 2}} } \right) \\ & \times Exposure\,time\left( {seconds} \right) \\ \end{aligned} $$

### Microbial reduction of prebagged human plasma (100 and 300 mL volumes) using 405-nm light

To assess microbial reduction, exposures were first conducted using 100 mL prebagged human plasma to demonstrate the broad-spectrum efficacy. To reflect a more clinically realistic volume, tests were then scaled up to treat 300 mL volumes of prebagged human plasma, spiked with *S. aureus*, *E. coli* and *C. albicans*. In each case, the prebagged seeded plasma was treated with ~ 16 mW cm^−2^ 405-nm light for up to 7 h (≤ 403 J cm^−2^), with 10 mL plasma held in identical conditions but foil-covered (dark control). The temperature of human plasma was monitored throughout the exposure period using a thermocouple, to ensure human plasma remained at 22 °C during treatment. Post-exposure, plasma samples were plated using nutrient agar, with the exception of *S. epidermidis* which was plated on tryptone soya agar, and malt extract agar with 0.1% yeast extract for *C. albicans*, and incubated at 37 °C for 24 h. Surviving microorganisms were enumerated with results recorded as mean microbial load (CFU mL^−1^, n ≥ 4 ± SD).

### Plasma protein compatibility of 300 mL light-treated prebagged plasma

#### Assessing post-exposure oxidative stress levels in human plasma using AOPP assay

An advanced oxidation protein products (AOPP) assay, which uses Chloramine-T as a marker of oxidative protein damage, was conducted on the plasma. The AOPP assay kit (ab242295; Abcam), was used in accordance with manufacturer’s instructions. In summary, treated and non-treated plasma samples were diluted in PBS (1:10). 200 µL diluted plasma (n = 3), and standards of Chloramine-T (0–100-µM), were transferred to a 96-well plate and 10 µL of Chloramine Reaction Initiator was added to all wells. The plate was shaken for 5 min (120 rpm, room temperature) and 20 µL Stop Solution was added. Absorbance at 340 nm was measured, and AOPP levels expressed as µM of Chloramine-T equivalents, with values corrected for dilution factor.

#### Assessing post-exposure clotting functionality using PTT and APTT assays

The stability of clotting factors in treated and non-treated plasma was measured using Prothrombin Time Test (PTT; AlphaLabs (UK)) and Activated Partial Thromboplastin Test (APTT; Enzyme Research Laboratories (UK)) assays, to assess the functionality of extrinsic-common and intrinsic-common coagulation cascades, respectively. Methodology was followed as per manufacturer’s instructions. Tests (n = 3) were conducted with time to clot manually recorded in seconds using a stopwatch.

#### Determination of post-exposure fibrinogen and Protein S levels

Human Fibrinogen SimpleStep (ab241383, Abcam) and Human Protein S (ab125969, Abcam) enzyme-linked immunosorbent assays (ELISAs) were used to quantitatively measure fibrinogen and Protein S content in plasma, respectively. Treated and non-treated plasma samples (n = 3) were serially diluted in Sample Diluent (1:100,000) prior to testing. ELISAs were used in accordance with manufacturer’s instructions. The concentration of target protein in human plasma samples was calculated using the standard curve with values corrected for dilution factor.

## Results

### Microbial reduction in prebagged human plasma

**Fig. 2:**
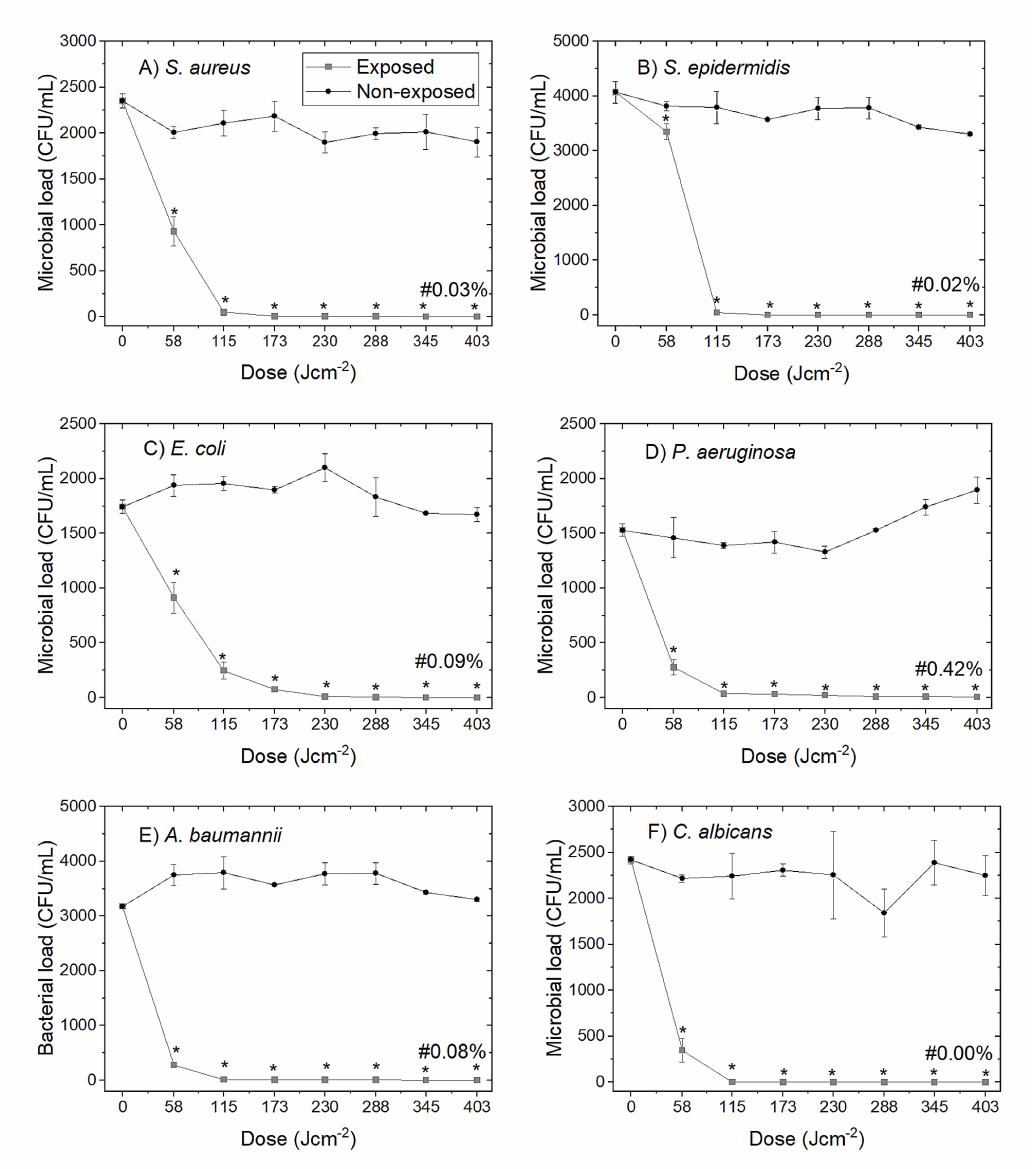
Broad-spectrum microbial reduction of 100 mL prebagged plasma using 405-nm light, as a function of dose. 100 mL prebagged human plasma seeded with contamination at ∼10^3^ CFU mL^−1^ (**A ***S. aureus*; **B ***S. epidermidis*; **C ***E. coli*; **D ***P. aeruginosa*; **E ***A. baumannii* and **F ***C. albicans*) was treated with16 mW cm^−2^ 405-nm light, under agitation (84 rpm; 22 °C), Data represents mean microbial load in CFU mL^−1^ (n =  ≥ 3, ± SD) with (*) representing a significant decrease in the microbial load in treated plasma when compared to the equivalent non-treated control [P ≤ 0.05; 2-sample *t*-test (Minitab v18)]. (#) represents the % survival after exposure to 403 J cm^−2^

Figure 2 illustrates the efficacy of 16 mW cm^−2^ 405-nm light for inactivation of A) *S. aureus*, B) *S. epidermidis*, C) *E. coli*, D) *P. aeruginosa*, E) *A. baumannii* and F) *C. albicans* contamination at approximately 10^3^ CFU mL^−1^ in 100-mL prebagged human plasma. All organisms were significantly reduced (P < 0.05) after exposure to an initial dose of 58 J cm^−2^. Inactivation kinetics of *S. aureus*, *S. epidermidis, P. aeuroginosa*, *A. baumannii* and *C. albicans* were similar, with most of the inactivation (> 97%) achieved after exposure to 115 J cm^−2^ (P = 0.032, 0.036, 0.035, 0.010 and 0.00, respectively). Contamination levels continued to decrease for the remainder of the exposure period with near-complete inactivation (≤ 10 CFU mL^−1^) observed following exposure to 288 J cm^−2^. The reduction of *E. coli* followed a similar trend but required slightly increased doses, with > 95% inactivation by 173 J cm^−2^.

**Fig. 3:**
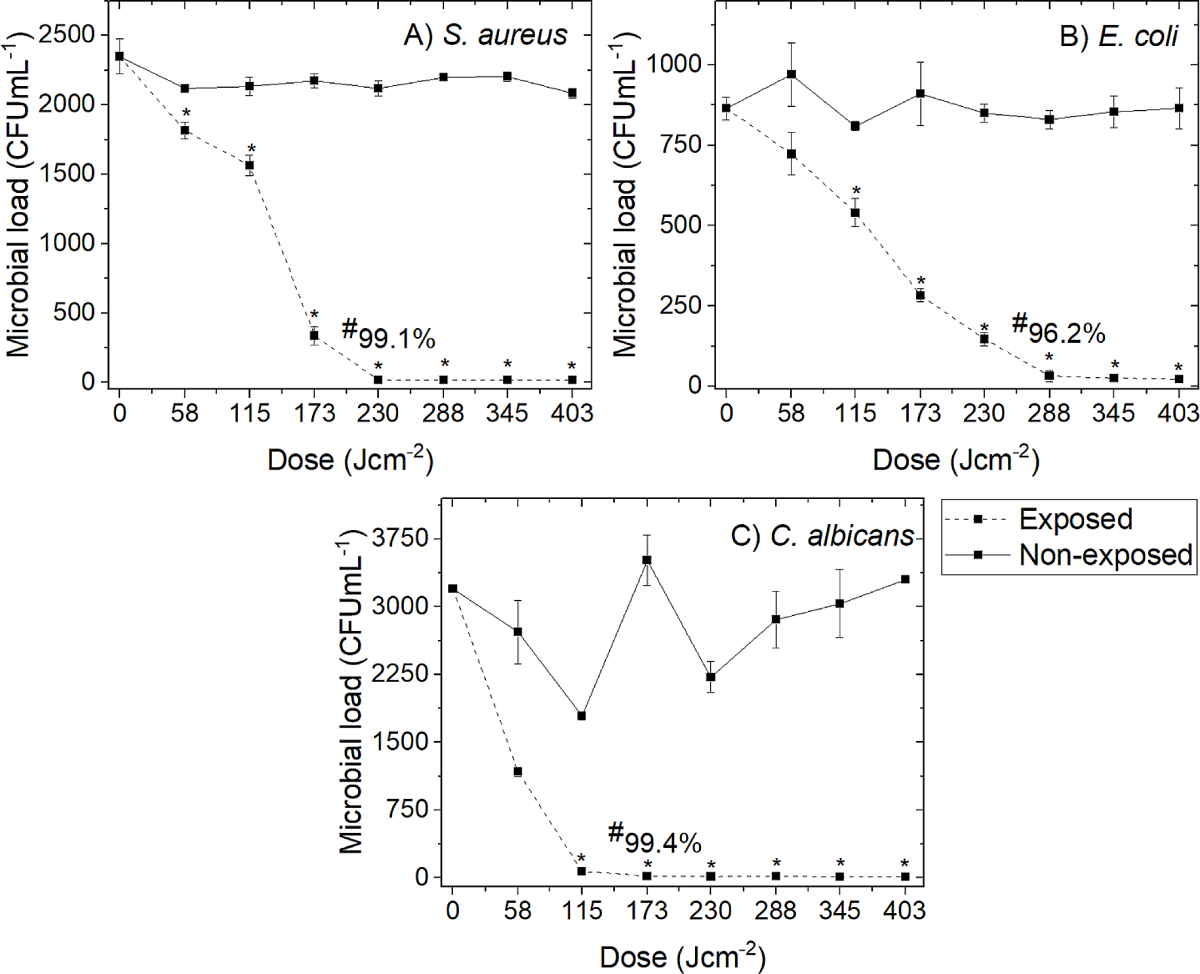
Microbial reduction of 300 mL prebagged human plasma using 405-nm light treatment, as a function of dose. Whole-bag plasma was spiked with **A ***S. aureus*, **B ***E. coli* and **C ***C. albicans* at approx. 10^3^ CFU mL^−1^ and exposed to 16 mW cm^−2^ under constant agitation (84 rpm; 22 °C). Data represents mean CFU mL^−1^ (n ≥ 4 ± SD), with (*) representing significant differences between treated and non-treated samples [P =  < 0.05; paired *t*-test (Minitab v18)]. (#) notes the % microbial reduction achieved by **A** 230 J cm^−2^, **B** 288 J cm^−2^ and **C** 173 J cm^−2^

Results from the exposure of 300 mL prebagged human plasma seeded with *S. aureus*, *E. coli* and *C. albicans* (approx. 10^3^ CFU mL^−1^) to 16 mW cm^−2^ 405-nm light are presented in Fig. 3. Of the organisms tested, *C. albicans* was the most susceptible to inactivation with 99.4% reduction achieved by 173 J cm^−2^ (P = 0.035). Inactivation kinetics for bacteria showed *S. aureus* to be reduced by 99.1% with 230 J cm^−2^ and 96.2% reduction of *E. coli* by 288 J cm^−2^ (P = 0.012 and 0.018 respectively).

### Assessing post-exposure oxidative stress using AOPP assay

**Fig. 4:**
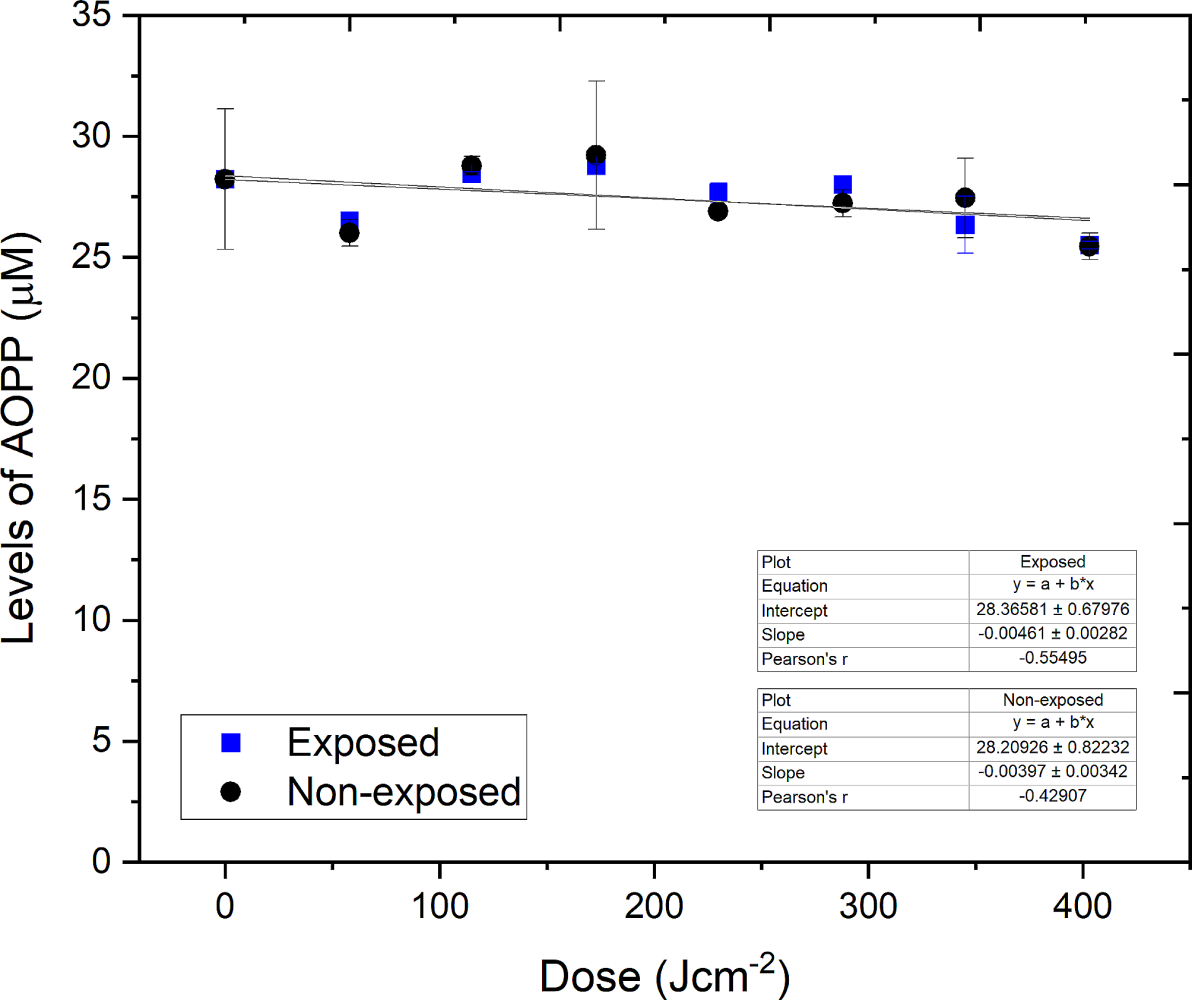
Levels of advanced oxidation protein products (AOPPs) detected in 300 mL prebagged plasma following exposure to 16 mW cm^−2^ 405-nm light (≤ 403 J cm^−2^). The concentration of AOPPs is expressed as µM of Chloramine-T equivalents, marker of oxidative damage, with values corrected for dilution factor. Data represent mean (n = 3 ± SD). No significant changes in AOPP levels were detected in treated plasma when compared to the equivalent non-treated control [P > 0.05; 2-sample *t* test (Minitab v18)]

Results (Fig. 4) show that there was no significant difference in the AOPP levels, indicative of general oxidative stress, between treated and non-treated plasma samples following exposure to the maximum applied dose of 403 J cm^−2^ (P > 0.05). The levels of AOPP of treated and non-treated samples fluctuated within the region of 25.5–29.2 µM, likely due to natural sample-to-sample variation in fibrinogen content, over the treatment period. AOPP levels in non-treated control plasma remained relatively constant (P = 0.371). These results suggest that 405 nm light doses up to 403 J cm^−2^ do not induce oxidative protein damage in treated plasma.

### Assessing post-treatment clotting functionality using PTT and APTT assays

**Fig. 5:**
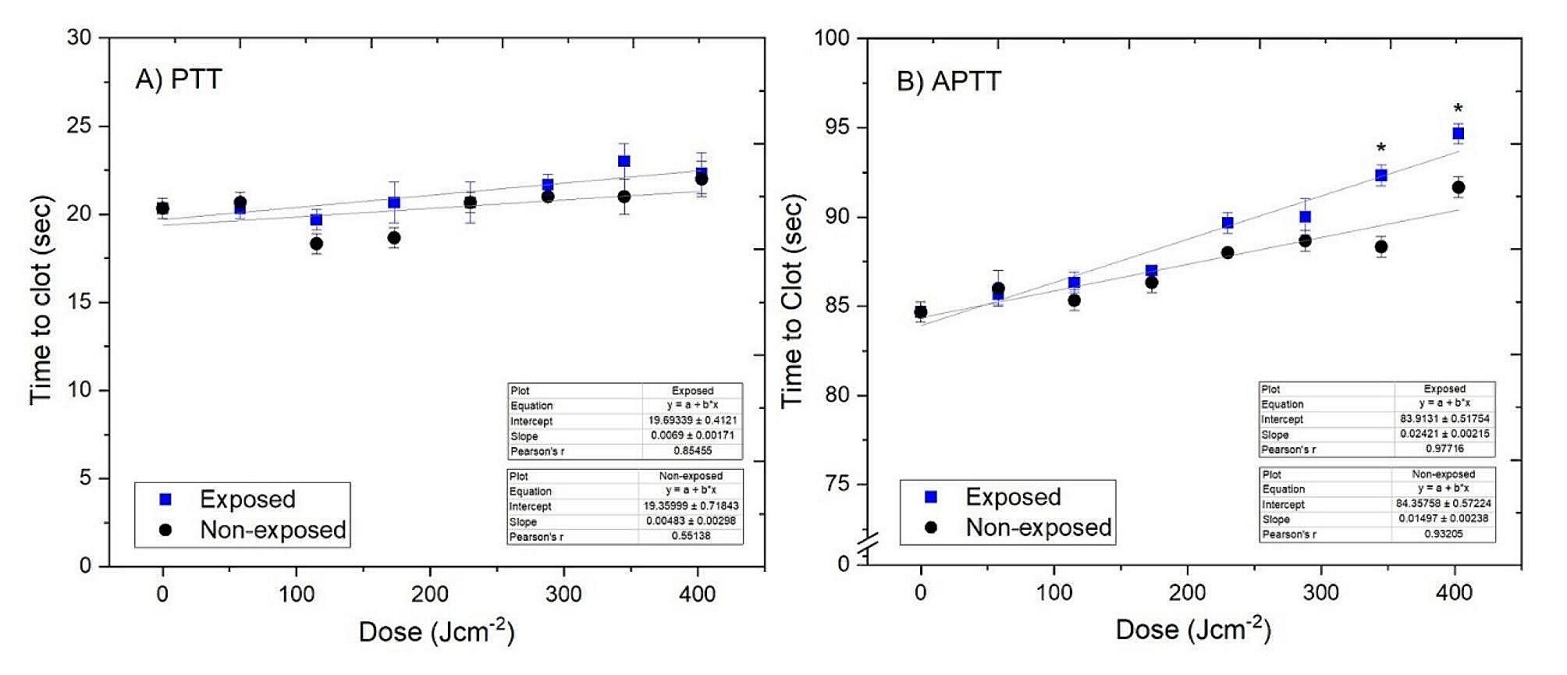
Assessment of the clotting functionality of 300 mL prebagged plasma treated with 405-nm light treated prebagged human plasma using **A** PTT and **B** APTT assays. Time to clot for human plasma treated with 16 mW cm^−2^ 405 nm light for doses up to 403 J cm^−2^ (7 h) compared to non-treated controls. Data represents mean time to clot (n = 3 ± SD) with (*) representing significant increase in clotting times between treated and non-treated human plasma [P ≤ 0.05; 2-sample *t*-test (Minitab v18)]

PTT and APTT assays were performed to assess the stability of extrinsic-common and intrinsic-common coagulation pathways, respectively. Figure 5A shows that there was no significant difference in PTT values between 300 mL prebagged human plasma treated with doses up to 403 J cm^−2^ and non-treated controls (P > 0.05). Results from the APTT assay (Fig. 5B), demonstrate that clotting times for 300 mL prebagged human plasma treated with doses up to 288 J cm^−2^ are similar to non-treated controls, with APTT values remaining relatively stable between 84 and 90 s (P > 0.05). A slight increase in APTT values was detected in human plasma treated to doses ≥ 345 J cm^−2^, with clotting times prolonged by a maximum of 4.3% compared to non-treated controls. These results suggest that exposure to 405 nm light doses up to 403 J cm^−2^ have little to no effect on the coagulation properties of prebagged human plasma. In general, trends from clotting assays showed slight increases in clotting times over the 7 h treatment period in both treated and non-treated samples, however the change was insignificant in both cases (P > 0.05).

### Determination of post-exposure fibrinogen and Protein S levels

**Fig. 6:**
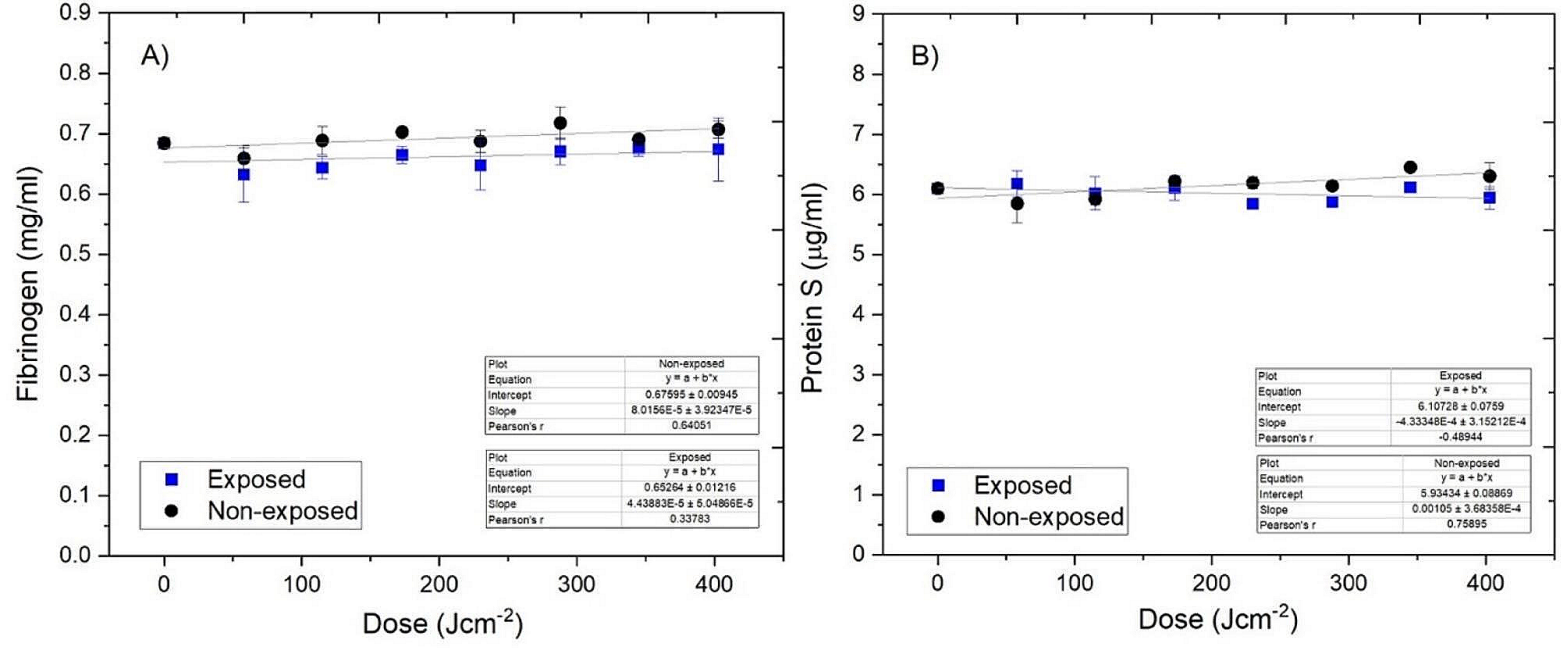
Fibrinogen (**A**) and Protein S (**B**) levels in 405 nm light treated 300 mL prebagged human plasma (≤ 403 J cm^−2^). Data represents the mean concentration of fibrinogen in human plasma (n = 3 ± SD). No significant differences were detected between treated and non-treated controls [P > 0.05; 2-sample *t* test (Minitab v18)]

Results from the fibrinogen and Protein S assays are shown in Fig. 6A and B, respectively. Minimal reductions were observed throughout the exposure period, with a maximum reduction of 6.5% for fibrinogen, after 288 J cm^−2^ (P = 0.306), and 5.7% for Protein S, after 403 J cm^−2^ (P = 0.326). Nevertheless, no change in fibrinogen or Protein S content was found to be significant at any point throughout the exposure period (P > 0.05). Fibrinogen content was relatively stable throughout the treatment period, with slight variations in the region of 0.63–0.67 mg mL^−1^ and 0.65–0.71 mg mL^−1^ for treated and non-treated human plasma, respectively. Protein S content slightly varied between 5.84–6.17 μg mL^−1^ and 5.85–6.46 μg mL^−1^ for treated and non-treated human plasma throughout the exposure period, respectively.

## Discussion

It is essential that a pathogen reduction treatment is capable of broad-spectrum antimicrobial efficacy and that these antimicrobial doses do not significantly impact the safety, quality, or effectiveness of the treated blood components. Results demonstrating the compatibility of 405-nm light as a microbicidal treatment for prebagged human plasma are described for the first time in this paper, with demonstration of the ability to reduce contamination in prebagged plasma up to volumes of 300 mL, and a range of proteomics tests assessing the stability and functionality of 405-nm light treated plasma. With microbial contamination being an infectious risk of *ex vivo* human platelet concentrates (PCs) stored in plasma at room temperature, this pilot study provides strong evidence of the broad-spectrum antibacterial efficacy for advancing to trials utilising large volume platelet concentrates.

Preliminary compatibility studies in an earlier study indicated that low volume (250 µL) human plasma samples treated with an effective microbicidal dose of 360 J cm^−2^ showed no signs of protein degradation via gel electrophoresis analysis and advanced oxidation protein products (AOPP) assay (Stewart et al. 2020). In the present, significantly scaled-up study, further positive results were obtained, with no signs of oxidative protein damage detected via the AOPP assay in prebagged 300-mL volumes of human plasma treated with light doses up to 403 J cm^−2^. It was expected that post-treatment fibrinogen levels would be unaffected since AOPP levels are a strong indicator for the stability of fibrinogen (Selmeci et al. 2006). Results from the fibrinogen ELISA confirmed this, with no significant changes in fibrinogen levels detected following exposure to doses up to 403 J cm^−2^. From these results, it appears that visible 405 nm light has a less adverse effect on the integrity of fibrinogen compared to UV-light, even when over 65× the dose is applied (403 J cm^−2^ 405 nm light versus 6 J cm^−2^ UV-light), with reports stating that UV-light based PRTs can potentially reduce fibrinogen in human plasma by up to 21% (Hornsey et al. 2009). The level of Protein S, an essential anti-clotting agent, was unaffected following exposure to antimicrobial doses up to 403 J cm^−2^ at > 94%, is similar to that of plasma treated with commercially available PRTs (Bubinski et al. 2021; Hornsey et al. 2009). The results of this study are also supportive of those in a recent study by Jackson et al (2024) which assessed the activities of a range of other coagulation factors (FV, FVII, FVIII, FIX, FX, FXI) in PCs and platelet poor plasma (PPP) using a dose of 270 J cm^−2^. The study demonstrated that 405 nm light exposure did not drastically effect activity of coagulation factors in PCs, but in some cases, differences were more notable in PPP, suggesting possible interplay between platelet surface and coagulation factors yields a protective effect on factor functions (Jackson et al 2024).

This study also provides further proof-of-concept results for the broad-spectrum microbicidal efficacy of 405-nm light for treatment of 300 mL prebagged human plasma, with near-complete inactivation (≤ 10 CFU mL^−1^) of the three representative organisms (Gram+ and − bacteria, and a yeast). Further, a wider panel of organisms tested in 100 mL prebagged volumes (Fig. 2) also demonstrated similar inactivation, supporting results of previous studies using lower volume, 250 µL, plasma samples (Stewart et al. 2020). As the transmissibility of 405-nm light in platelets stored in plasma is within the same region as human plasma, at 0.1–0.3% compared to 0.38–0.70% (based on data from this study and (Maclean et al. 2020)), we envision that similar microbial reductions would be observed in prebagged platelets stored in plasma.

The previous small-scale study by Stewart et al (2020) demonstrated that 405-nm light was capable of inactivating bacteria seeded at a range of densities (10^1^–10^8^ CFU mL^−1^) in small volume (250 µL) human plasma samples, with a fixed dose of 360 J cm^−2^ achieving 95.1–100% inactivation across all contamination levels. As naturally occurring levels of bacterial contamination in blood products are typically low, ranging from 10 to 100 cells per unit prior to storage, low level contamination at approximately 10^3^ CFU mL^−1^ was selected for investigation in the present study (Figs. 2, 3) to reflect a realistic clinical scenario in large prebagged volumes (Hillyer et al. 2003).

Whilst these dose levels are relatively high compared to existing UV-light based technologies (typically delivering doses in the region of 3 J cm^−2^) treatment by 405 nm light eliminates the need for additive photosensitive agents, which lengthens the processing times required to remove the additive chemicals to reduce the risk of adverse reaction in recipients (Irsch and Lin 2011; Liu and Wang 2021).

To treat or prevent bleeding in patients, it is important to ensure that an antimicrobial treatment of blood transfusion products has little to no effect on the stability and functionality of clotting factors. Prothrombin Time (PTT) and Activated Partial Thromboplastin Time (APTT) tests were used to assess potential changes in coagulation activity in 405-nm light treated human plasma. Analysis of PTT results (Fig. 5a) indicates that 405-nm light has minimal effect on clotting factors involved in the extrinsic (factor VII) and common coagulation (factors I, II, V and X), with no significant differences in time to clot detected between treated and non-treated human plasma following exposure to doses up to 403 J cm^−2^ (P > 0.05). The time to clot, measured via the APTT assay, was slightly higher in human plasma treated with 405-nm light doses ≥ 345 J cm^−2^, suggesting that intrinsic clotting factors (factors VIII, IX, XI, and XII) may be more photo-sensitive compared to extrinsic and common coagulation pathway factors. Nevertheless, the overall impact on clotting activity, with a maximum prolongation of 4.3% over the treatment period, is relatively low in comparison to clinically approved, UV-light based PRTs that have shown to prolong clotting times by up to 24% (Hornsey et al. 2009).

In this report, microbial inactivation and compatibility studies were conducted using a fixed 405 nm light treatment using an irradiance of 16 mW cm^−2^, however previous studies have demonstrated antimicrobial efficacy in human plasma using a range of irradiances between 5 and 100 mW cm^−2^ (Maclean et al. 2016; Stewart et al 2020). Research has shown that use of lower irradiances is more germicidally energy efficient compared to higher irradiances for pathogen reduction of human plasma and platelets stored in plasma. This is thought to be due to a threshold level of photons that can interact with porphyrins at any one time, referred to as the as the free porphyrin to photon ratio (Maclean et al 2020; Maclean 2016). Whilst an important consideration, the method of dose delivery must be selected in line with the application type, as the irradiance level directly influences the exposure time required to apply an effective antimicrobial dose. It is envisioned that the method of dose delivery may be adjusted to suit the practical application, as per Eq. 1, i.e. utilising higher irradiances (and therefore shorter treatment times) for rapid decontamination pre or post-storage, or lower irradiances to continuously irradiate prebagged plasma or platelets stored in plasma during the inventory period in hospitals. Nevertheless, future work is required to assess how varying the dose delivery regime may impact the compatibility of 405-nm light with human plasma, and platelet concentrates suspended in plasma.

In conclusion, these results indicate that effective antimicrobial light doses up to 403 J cm^−2^ cause little to no changes to protein stability or *in vitro* functionality of the factors tested in this study (fibrinogen, Protein S, AOPP, PTT, APTT). This indicates the potential for doses in the region of 270 J cm^−2^, previously shown to be capable of viral and parasitic inactivation in human plasma (4-log and 9-log reductions respectively), to be applied without comprising plasma quality (Jankowska et al. 2020; Ragupathy et al. 2022). Further, 405-nm light has also shown potential compatibility with human platelets stored in plasma, a more sensitive cellular blood component, using antimicrobial doses up to 288 J cm^−2^, with the recovery of treated and non-treated platelets shown to be statically similar in a murine model (P > 0.05) (Maclean et al. 2020). This study, together with previous results, provides further evidence supporting the potential compatibility of antimicrobial doses of 405-nm light for treatment of plasma.

## Data Availability

Data supporting this publication are stored by the University of Strathclyde. Details of the data and how it can be accessed are available from the University of Strathclyde KnowledgeBase at 10.15129/83df4ab2-e0cb-40ff-8ebb-62792a31c16d

## Abbreviations

*C. albicans*: *Candida albicans*
CFU mL^−^^1^: Colony-forming units per millilitre
*E. coli*: *Escherichia coli*
FWHM: Full-width half-maximum
PCs: Platelet concentrates
PRTs: Pathogen reduction technologies
ROS: Reactive oxygen species
*S. aureus*: *Staphylococcus aureus*
UV: Ultraviolet

## References

[CR1] Brecher ME, Hay SN, Rothenberg SJ (2003). Monitoring of apheresis platelet bacterial contamination with an automated liquid culture system: a university experience. Transfusion.

[CR2] Bubinski M, Gronowska A, Szykula P, Kluska K, Kuleta I, Ciesielska E, Picard-Maureau M, Lachert E (2021). Plasma pooling in combination with amotosalen/UVA pathogen inactivation to increase standardisation and safety of therapeutic plasma units. Transfus Med.

[CR4] Enwemeka CS, Williams D, Hollosi S, Yens D, Enwemeka SK (2008). Visible 405 nm SLD light photo-destroys methicillin-resistant *Staphylococcus aureus* (MRSA) in vitro. Laser Surg Med.

[CR5] Escolar G, Diaz-Ricart M, McCullough J (2022). Impact of different pathogen reduction technologies on the biochemistry, function, and clinical effectiveness of platelet concentrates: an updated view during a pandemic. Transfusion.

[CR6] Gehrie EA, Rutter SJ, Snyder EL (2019). Pathogen reduction: the state of the science in 2019. Hematol Oncol Clin North Am.

[CR7] Godley BF, Shamsi FA, Liang FQ, Jarrett SG, Davies S, Boulton M (2005). Blue light induces mitochondrial DNA damage and free radical production in epithelial cells. J Biol Chem.

[CR8] Guffey SJ, Wilborn J (2006). In vitro bactericidal effects of 405-nm and 470-nm blue light. Photomed Laser Surg.

[CR9] Hamblin MR, Viveiros J, Yang C, Ahmadi A, Ganz RA, Tolkoff MJ (2005). *Helicobacter pylori* accumulates photoactive porphyrins and is killed by visible light. Antimicrob Agents Chemother.

[CR10] Hamzah N, Karim FA, Ahmad AH, Yusof NM (2018). Effects of methylene blue, psoralen and riboflavin treatments on fibrinogen and other coagulation factors level of fresh frozen plasma. Mal J Med Health Sci.

[CR11] Hillyer CD, Josephson CD, Blajchman MA, Vostal JG, Epstein JS, Goodman JL (2003). Bacterial contamination of blood components: risks, strategies, and regulation: joint ASH and AABB educational session in transfusion medicine. Hematol Am Soc Hematol Educ Program.

[CR12] Hornsey VS, Drummond O, Morrison A, McMillan L, MacGregor IR, Prowse CV (2009). Pathogen reduction of fresh plasma using riboflavin and ultraviolet light: effects on plasma coagulation proteins. Transfusion.

[CR13] Irsch J, Lin L (2011). Pathogen inactivation of platelet and plasma blood components for transfusion using the INTERCEPT blood system™. Transfus Med Hemother.

[CR14] Jackson JW, Kaldhone PR, Parunov LA, Stewart CF, Anderson JG, MacGregor SJ, Maclean M, Atreya CD (2024). Human platelet concentrates treated with microbicidal 405 nm light retain hemostasis activity. J Photochem Photobiol B.

[CR15] Jankowska KI, Nagarkatti R, Acharyya N, Dahiya N, Stewart CF, Macpherson RW, Wilson MP, Anderson JG, MacGregor SJ, Maclean M, Dey N, Debrabant A, Atreya CD (2020). Complete inactivation of blood borne pathogen *Trypanosoma cruzi* in stored human platelet concentrates and plasma treated with 405 nm violet-blue light. Front Med.

[CR16] Kaldhone PR, Azodi N, Markle HL, Dahiya N, Stewart C, Anderson J, MacGregor S, Maclean M, Nakhasi HL, Gannavaram S, Atreya C (2024). The preclinical validation of 405 nm light parasiticidal efficacy on *Leishmania donovani* in ex vivo platelets in a Rag2−/− mouse model. Microorganisms.

[CR17] Klein HG (2005). Pathogen inactivation technology: cleansing the blood supply. J Intern Med.

[CR18] Liu H, Wang X (2021). Pathogen reduction technology for blood component: a promising solution for prevention of emerging infectious disease and bacterial contamination in blood transfusion services. J Photochem Photobiol.

[CR19] Lu M, Dai T, Hu S, Zhang Q, Bhayana B, Wang L, Wu MX (2020). Antimicrobial blue light for decontamination of platelets during storage. J Biophotonics.

[CR20] Maclean M, MacGregor SJ, Anderson JG, Woolsey G (2009). Inactivation of bacterial pathogens following exposure to light from a 405-nanometer light-emitting diode array. Appl Environ Microbiol.

[CR21] Maclean M, Murdoch LE, MacGregor SJ, Anderson JG (2013). Sporicidal effects of high-intensity 405 nm visible light on endospore-forming bacteria. Photochem Photobiol.

[CR22] Maclean M, Anderson JG, MacGregor SJ, White TA, Atreya CD (2016). A new proof of concept in bacterial reduction: antimicrobial action of violet-blue light (405 nm) in ex vivo stored plasma. J Blood Transfus.

[CR23] Maclean M, Gelderman MP, Kulkarni S, Tomb RM, Stewart CF, Anderson JG, MacGregor SJ, Atreya CD (2020). Non-ionizing 405 nm light as a potential bactericidal technology for platelet safety: evaluation of in vitro bacterial inactivation and in vivo platelet recovery in severe combined immunodeficient mice. Front Med.

[CR24] Magron A, Laugier J, Provost P, Boilard E (2018). Pathogen reduction technologies: the pros and cons for platelet transfusion. Platelets.

[CR25] Marschner S, Dimberg LY, Shaz BH, Hillyer CD, Gil MR (2019). Chapter 48—pathogen reduction technologies. Transfusion medicine and hemostasis.

[CR26] McKenzie K, Maclean M, Grant MH, Ramakrishnan P, Macgregor SJ, Anderson JG (2016). The effects of 405 nm light on bacterial membrane integrity determined by salt and bile tolerance assays, leakage of UV-absorbing material and SYTOX green labelling. Microbiology.

[CR27] Murdoch LE, Maclean M, Endarko E, MacGregor SJ, Anderson JG (2012). Bactericidal effects of 405-nm light exposure demonstrated by inactivation of *Escherichia*, *Salmonella*, *Shigella*, *Listeria* and *Mycobacterium* species in liquid suspensions and on exposed surfaces. Sci World J.

[CR28] Murdoch LE, McKenzie K, Maclean M, MacGregor SJ, Anderson JG (2013). Lethal effects of high-intensity violet 405-nm light on *Saccharomyces cerevisiae, Candida albicans,* and on dormant and germinating spores of *Aspergillus niger*. Fungal Biol.

[CR29] Ragupathy V, Haleyurgirisetty M, Dahiya N, Stewart C, Anderson J, MacGregor S, Maclean M, Hewlett I, Atreya C (2022). Visible 405 nm violet-blue light successfully inactivates HIV-1 in human plasma. Pathogens.

[CR30] Ramakrishnan P, Maclean M, MacGregor SJ, Anderson JG, Grant MH (2016). Cytotoxic responses to 405 nm light exposure in mammalian and bacterial cells: involvement of reactive oxygen species. Toxicol In Vitro.

[CR31] Schlenke P (2014). Pathogen inactivation technologies for cellular blood components: an update. Transfus Med Hemother.

[CR32] Selmeci L, Székely M, Soós P, Seres L, Klinga N, Geiger A, Acsády G (2006). Human blood plasma advanced oxidation protein products (AOPP) correlates with fibrinogen levels. Free Radic Res.

[CR33] Seltsam A (2017). Pathogen inactivation of cellular blood products—an additional safety layer in transfusion medicine. Front Med.

[CR34] Stewart CF, Tomb RM, Ralston HJ, Armstrong J, Anderson JG, MacGregor SJ, Atreya CD, Maclean M (2020). Violet-blue 405-nm light-based photoinactivation for pathogen reduction of human plasma provides broad antibacterial efficacy without visible degradation of plasma proteins. Photochem Photobiol.

[CR35] Tomb RM, Maclean M, Coia JE, Graham E, McDonald M, Atreya CD, MacGregor SJ, Anderson JG (2017). New proof-of-concept in viral inactivation: virucidal efficacy of 405 nm light against feline calicivirus as a model for norovirus decontamination. Food Environ Virol.

[CR36] Tomb RM, White TA, Coia JE, Anderson JG, MacGregor SJ, Maclean M (2018). Review of the comparative susceptibility of microbial species to photoinactivation using 380–480 nm violet-blue light. Photochem Photobiol.

